# Genetic variations in *ATM* and *H2AX* loci contribute to risk of hematological abnormalities in individuals exposed to BTEX chemicals

**DOI:** 10.1002/jcla.24321

**Published:** 2022-03-02

**Authors:** Samaneh Jafari Roshan, Yaser Mansoori, Seyed Reza Hosseini, Davood Sabour, Abdolreza Daraei

**Affiliations:** ^1^ Student Research Committee Babol University of Medical Sciences Babol Iran; ^2^ Department of Medical Genetics School of Medicine Babol University of Medical Sciences Babol Iran; ^3^ Noncommunicable Diseases Research Center Fasa University of Medical Sciences Fasa Iran; ^4^ Social Determinants of Health Research Center Health Research Institute Babol University of Medical Sciences Babol Iran; ^5^ Cellular and Molecular Biology Research Center Health Research Institute Babol University of Medical Sciences Babol Iran

**Keywords:** *ATM*‐rs228589 A>T, BTEXs, *H2AX*‐rs7759 A>G, hematotoxicity, *WRN*‐rs1800392 G>T

## Abstract

**Background:**

Loci controlling DNA double‐strand breaks (DSBs) repair play an important role in defending against the harmful health effects of benzene, toluene, ethylbenzene, and xylene (BTEX), but their gene variants may alter their repair capacity. The aim of the current study was to determine the relationship of functional polymorphisms ATM‐rs228589 A>T, WRN‐rs1800392 G>T and H2AX‐rs7759 A>G in DBS repair loci with the abnormal hematological indices in workers who exposed to BTEXs.

**Methods:**

We included 141 cases with one or more abnormal hematological parameters, who had been occupationally exposed to BTEX chemicals and 152 controls with a similar exposure condition but without any abnormal hematological parameters. Atmospheric concentrations of BTEXs were measured and whole blood samples were taken from the participants to determine hematologic parameters and SNP genotyping.

**Results:**

Results showed that T allele of ATM‐rs228589 and G allele of H2AX‐rs7759 had a higher frequency in cases than controls (*p* = 0.012 and *p* = 0.001, respectively). Also, AT and TT genotypes of ATM‐rs228589 and AG and GG genotypes of H2AX‐rs7759 were higher in cases compared to controls. The AT and TT genotypes of ATM‐rs228589 have significant associations with a risk of hematological abnormalities in the codominant (AT vs. AA, *p* = 0.018), dominant (AT + TT vs. AA, *p* = 0.010) and overdominant (AT vs. AA + TT, *p* = 0.037) models. The GG and AG genotypes of H2AX‐rs7759 were in relation with increased risk of abnormal hematological indices under codominant (GA vs. AA, *p* = 0.009 & GG vs. AA, *p* = 0.005), dominant (AG + GG vs. AA, *p* = 0.001), and recessive (GG vs. AA + AG, *p* = 0.025) models.

**Conclusions:**

These observations may help to understand the mechanisms of BTEX hematotoxicity and identify useful biomarkers of risk assessment for workers exposed to BTEX.

## Graphical abstract

Our results demonstrated that T allele of ATM‐rs228589 and G allele of H2AX‐rs7759 were significantly associated with the risk of BTEX‐related hematotoxicity in an Iranian subpopulation (*p* = 0.012 and *p* = 0.001, respectively). Additionally, we observed an association of reduced RBC counts with the T allele of ATM‐rs228589 (*p*‐trend = 0.010), while increased granulocyte counts and decreased lymphocyte counts were associated with the G allele of H2AX‐rs7759 (*p*‐trend = 0.008 and 0.010, respectively).

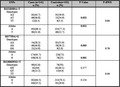

## 1 INTRODUCTION

Benzene, toluene, ethylbenzene, and xylene (BTEX) chemicals belong to a group of toxic volatile organic compounds (VOCs) that cause serious damage to human health.^1^, ^2^ One of the leading sources for producing and releasing of BTEXs in the environment is the decomposition of some compounds including the gasoline, crude oil, and other fuels used in the petrochemical industries, which provide continuous exposure to high BTEX concentration for individuals.^1^, ^3^ Various harmful effects due to exposure to these chemicals can occur in different organs; the most prominent of which are severe cellular abnormalities of the bone marrow and hematopoietic system, including anemia, leukopenia, thrombocytopenia, pancytopenia, and leukemia.^1^, ^4^, ^5^, ^6^, ^7^ Because these compounds, especially benzene, act as a hemotoxin, leukemogen, as well as carcinogen.^4^, ^6^, ^8^, ^9^ Although the molecular mechanisms by which BTEXs lead to hematotoxic defects are not yet fully revealed, it has been indicated that these chemicals are genotoxic through triggering genetic aberrations at both chromosomal and gene levels.^2^, ^10^, ^11^ It has also been demonstrated that their genotoxic mechanisms are mediated by DNA oxidative damage through the generation of reactive oxygen species (ROSs).^2^ The most severe toxic form of DNA lesions caused by ROSs is DNA double‐strand breaks (DSBs) which induce genome instability, chromosome abnormalities, apoptosis, and cell cycle suppression.^12^, ^13^ In addition, there is evidence that hematopoietic progenitor cells in the bone marrow are more sensitive to DNA DSB lesions because of their high proliferation and differentiation.^13^ There are two main pathways for repairing the DSB lesions, including homologous recombination (HR) and non‐homologous end joining (NHEJ).^12^ One of the important genes controlling these two pathways is the ataxia telangiectasia mutated (*ATM*) gene, which encodes vital serine/threonine kinase involved in the maintenance of genomic integrity via activating cell cycle checkpoints and stimulating the repair of DNA damages. Following the occurrence of DNA DSBs, the ATM protein is activated and phosphorylates critical protein substrates, such as H2AX, to transduce a repair signal to downstream effectors.^14^, ^15^, ^16^ H2AX is a variant form of histone H2A, and the amount of its phosphorylated form (γH2AX) locally increases at the site of DSB via serine‐threonine kinases such as ATM.^17^, ^18^ The formation of γH2Ax is a key marker of DSB^18^, ^19^ and results in activating two main pathways of the DSB repair, including HR and NHEJ, depending on the phase of the cell cycle.^17^ The *WRN* is another important gene in the DSB repair pathway through encoding a protein belonging to the RecQ helicase family with helicase and exonuclease activities, which is responsible for retaining genome stability. Moreover, the WRN is necessary to interact with other functional proteins in an efficient repair pathway.^20^ Data also exist that *ATM*, *WRN*, and *H2AX* loci play key roles in protecting the genome against some toxins inducing DBSs.^13^, ^21^, ^22^ In addition, the mutation repair activities of these genes play a key role in the physiological function and genomic integrity of hematopoietic cells.^13^, ^18^, ^23^, ^24^ Interestingly, some occupational association studies on workers with similar BTEX exposure have provided evidence that individuals can be markedly different in their responses to the hematotoxicity effects of BTEXs due to the variability of genetic profiles, signifying a role of individual genetic susceptibility behind the BTEX effects.^13^, ^25^, ^26^, ^27^, ^28^

In the light of this evidence, we hypothesized that genetic variations within *ATM*, *WRN*, and *H2AX* loci could modify the double‐strand repair capacity of the individuals, and in turn, confer an individual genetic susceptibility to BTEX‐associated hematotoxicity. Revealing such a role of genetic predisposition in the BTEX responsiveness can be of great importance in early detection and chemical exposure risk assessment of susceptible individuals for exposure‐induced diseases.^29^, ^30^, ^31^ Therefore, the present cross‐sectional study was aimed to investigate the relationship of some functional single‐nucleotide polymorphisms (SNPs) within the *ATM*, *WRN*, and *H2AX* genes, including *ATM*‐rs228589 A>T, *WRN*‐rs1800392 G>T, and *H2AX*‐rs7759 A>G, with BTEX‐related hematological abnormalities in an Iranian occupational subpopulation.

## 2 MATERIALS AND METHODS

### 2.1 Study population and clinical sample collection

In this cross‐sectional study, we randomly selected 293 men participants who worked in a chemical industry unit in the south of Iran. Sample size was estimated based on the following equation: Sample size = (r + 1(p*)(1 − p*)(Z_ + Z(_/2))2)/r (p1 − p2)2, where, r = ratio of cases to controls, 1 for equal number of case and controls, p* = proportion of exposed cases + proportion of control exposed/2; Z_ = Standard normal variate for power/margin of error = for 80% power, it is 0.84; for 90%, it is 1.28, and for 95%, it is 1.96; Z_/2 = Standard normal variate for level of significance (at 5% type error, it is 1.96 and at 1% type error, it is 2.58); p1 = proportion in cases based on previous studies; p2 = proportion in controls based on previous studies. Hence, sample size = 1 + 1(0.375) (1 − 0.375) (1.96 + 1.96)2 1(0.50 − 0.25)2 = 2 (0.375) (0.625) (15.36) 0.0625 = 115.2. This clearly indicates that at least 115 individuals should be selected from each group.

All participants were manual workers that were continually in contact with BTEX components for at least 2 consecutive years and without hematological disorders such as glucose‐6‐phosphate dehydrogenase (G6PD) deficiency, hemophilia, minor thalassemia, and any history of exposure to other chemicals with hematotoxic effects. Moreover, chronic disorders, such as diabetes, dyslipidemia, hypertension, cardiovascular diseases, and obesity, were considered as exclusion criteria for the study population. The first phase of the study was proceeded by collecting blood samples of all subjects for measuring the complete blood count (CBC) parameters, including white blood cell count (WBC), WBC differential, red blood cell count (RBC), hemoglobin (HGB), hematocrit (HCT), mean corpuscular volume (MCV), mean corpuscular hemoglobin (MCH), mean corpuscular hemoglobin concentration (MCHC), red cell distribution width (RDW), and platelet (PLT) counts. In the second phase of the study and through the results of the CBC analysis, 141 subjects with one or more abnormal hematological indexes were defined as the case group, and 152 participants with normal hematological parameters were considered the control group. The individual characteristics of each participant were collected via interview and a questionnaire containing information of demographic variables, medical history, smoking habits, drug consumption, and occupation‐related data (job category, job history, working hours/day, length of exposure (employment), and occupational or non‐occupational exposure to other chemicals). The study was standardized with the Helsinki Declaration of 1964 protocol as revised in 2013^32^ and all subjects freely signed a consent form before starting the study. The study protocol was approved by the Ethics Committee of Babol University of Medical Sciences (ethical code: 9706614).

### 2.2 BTEX exposure assessment of the subjects via collection and analysis of BTEXs in the air environment

The sampling and analysis method of BTEXs for the exposure assessment were according to the National Institute for Occupational Safety and Health (NIOSH) protocol 1501^33^ using activated charcoal sorbent tubes and a sampling pump with a flow rate of 100–200 ml/min. Three samples of air from the breathing zone of each person were collected during different hours of working shifts. In the same way, a sample from the environment air at the breathing zone height was collected from each administrative office. The samples were delivered to the laboratory for analysis through gas chromatography with flame ionization detection according to the NIOSH technique.

### 2.3 SNP Selection, DNA extraction, and SNP genotyping assay

We used data from the 1000 Genomes Project and dbSNP (https://www.ncbi.nlm.nih.gov/) databases for selecting the SNPs of interest. The three important SNPs within *ATM*, *WRN*, and *H2AX* genes were candidates with key roles in the double‐strand DNA repair system, and our selection criteria were a minor allele frequency of >5% and clues of their functionality in experimental investigations. Target SNPs were *ATM*‐rs228589 A>T, *WRN*‐ rs1800392 G>T, and *H2AX*‐rs7759 A>G. The selected polymorphisms for this work had a MAF ≥ 0.3 and their single‐nucleotide change resulted in the gain or loss of a restriction site making them suitable for detection by PCR and the restriction fragment length polymorphism (RFLP) analysis (PCR‐RFLP). The DNA was isolated from peripheral blood leukocytes using a commercially available kit (Qiagen, Hilden, Germany). The extracted DNA samples were stocked up at −20°C until PCR. The concentration and purity of DNA were assessed using a NanoDrop spectrophotometer (Thermo Scientific). The PCR amplification was performed using the forward primer 5′‐TGTCGCTGTGTTTGCTTTAAC‐3′ and the reverse primer 5′‐GAACCTCCGAATGACGAAGAA‐3′ for rs228589 polymorphism of the *ATM* gene as well as the forward primer 5′‐GCATCCTGTTGGAGGAAGTA‐3′ and the reverse primer 5′‐TGCAGTTATCCACACTCACA‐3′ for rs7759 in *H2AX* gene and the forward primer 5′‐CCCACTGGGAATTTGAAGGT‐3′ and the reverse primer 5′‐ACTTCCACTATGAGCAACGG‐3′ for rs1800392 in *WRN* gene. Primer spanning each of the SNP variants was designed using the NCBI Primer‐BLAST tool (https://www.ncbi.nlm.nih.gov/tools/primer‐blast/). PCR reaction was conducted in 20 μl volume containing 12.5 μl of Taq DNA Polymerase 2x Master Mix (Ampliqon), 2 μl of each primer (5 pM), 3.5 μl sterile D.W and 2 μl DNA template (≤100 ng). The PCR thermocycler was performed as an initial denaturation at 94°C for 5 min following 36 cycles of 3 steps, including, 94°C for 30 s, 56°C for the *WRN* and *H2AX* for 30 s, and 60°C for the *ATM* for 35 s; the final extension was at 72°C for 35 s. The expected PCR fragment lengths were 258 bp, 219 bp, and 283 bp for the *ATM*, *WRN*, and *H2AX* genes, respectively.

**FIGURE 1:**
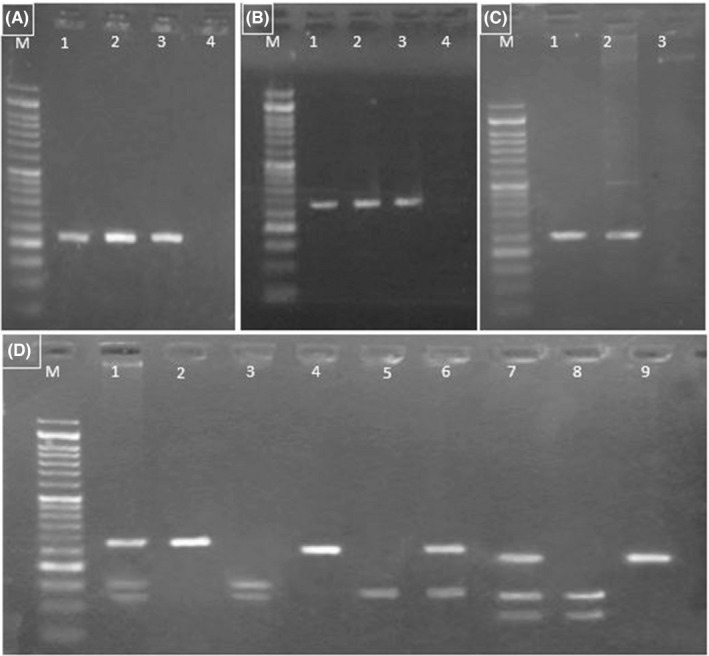
The PCR assay and agarose gel electrophoresis for genotyping of ATM‐RS228589 A>T, WRN‐RS1800392 G>T, and H2AX‐RS7759 A>G Polymorphisms. (A) M: 50bp DNA ladder; lanes 1–3: PCR products showing 219bp (WRN‐RS1800392 G>T); lane 4: negative control. (B) M: 50bp DNA ladder; lanes 1–3: PCR products showing 283bp (H2AX‐RS7759 A>G); lane 4: negative control. (C) M: 50bp DNA ladder; lanes 1–2: PCR products showing 258bp (ATM‐RS228589 A>T); lane 3: negative control. (D) The digested PCR products and corresponding different genotypes: From left to right: Lane M marker (50bp ladder); lanes 1–3 show three different genotypes of H2AX rs7759; lane 1 represents heterozygote A/G with 283, 157, and 126bp bands; lane 2 is homozygote A/A with single 283bp band; lane 3 is homozygote G/G with 157 and 126bp bands. Lanes 4–6 display genotypes for ATM rs228589: lane 4 represents homozygote A/A with single 258bp band; lane 5 represents homozygote T/T with 135 and 123bp bands; lane 6 shows heterozygote A/T with 258, 135 and 123bp bands. Lanes 7–9 indicate WRN rs1800392 genotypes: lane 7 represents heterozygote G/T with 219, 133, and 86bp bands; lane 8 represents homozygote T/T with 133 and 86bp bands; lane 9 represents homozygote G/G with single 219bp band

After amplification using a thermocycler PCR System, sizes of PCR products were confirmed through 2% agarose gel electrophoresis stained with a safe stain (Biofact). To determine the genotyping of SNPs using the RFLP analysis, PCR products containing SNPs were treated with specific restriction endonuclease enzymes. Details of the PCR‐RFLP assay, including the size of PCR products, restriction enzymes, recognition and cutting sequence site, and RFLP genotype patterns, are shown in Table 1. The amplified PCR products of the *WRN*‐rs1800392 G>T and *H2AX*‐rs7759 A>G were digested by the HinfI and Alw21I restriction enzymes (Thermo Scientific or Fermentas, Germany), respectively, at 37°C for 16 h. The restriction enzymes were deactivated at 65°C for 20 min. Regarding the *ATM*‐rs228589 A>T, PCR products were digested via BseGI restriction enzyme (Thermo Scientific) at 55°C for 16 h. At the end of the incubation time, the restriction enzyme was deactivated at 80°C for 20 min. The patterns of enzymatic digestions were visualized and analyzed on a 2% agarose gel stained with a safe stain (Biofact) under ultraviolet light (Figure 1). Finally, according to the genotype patterns, the related PCR products were randomly chosen for direct sequencing by the Sanger sequencing (Niagenes Company, Iran). The sequences of PCR products flanking the given SNPs were visualized by the CodonCode Aligner software.

**TABLE 1:** Details of RFLP‐PCR technique in genotyping of SNPs under investigation

Polymorphism	Enzyme	PCR product size	Recognition sequence and cutting site	Restriction fragments (bp)
ATM‐RS228589 A>T	BseGI	258	5′…G G A T G N N**↓**…3′	AA;258(no cut) AT;128+130+258 TT;128+130
H2AX‐RS7759 A>G	BsiHKAI	283	5′…G T G C T**↓**C…3′	AA;283(no cut) AG;126+157+283 GG;126+157
WRN‐RS1800392 G>T	HinfI	219	5′…G**↓**A N T C…3′	GG;219(no cut) GT;219+86+133 TT;86+133

Abbreviations: ATM, ataxia telangiectasia mutated; bp, base pair; PCR, polymerase chain reaction.

### 2.4 Statistical analysis

Microsoft Excel and Statistical Package for the Social Sciences (SPSS), version 22 (IBM Corp) were used to perform the statistical analyses. The normality of the distributions of data was assessed by the Kolmogorov–Smirnov test. When Kolmogorov–Smirnov test showed that dependent variables were normally distributed, the student *t* test was used to determine the statistical significance of the differences in quantitative variables and the chi‐square test was used for categorical variables. Continuous variables were defined as mean ± SD and categorical variables were defined as absolute and percentage values. The accordance of genotypes frequency with the Hardy–Weinberg equilibrium in case and control groups was determined by the chi‐square test. The difference between the allelic/genotypic frequency distribution was calculated using the *χ* ^2^ test. The association between the three polymorphisms and the risk of hematotoxicity was tested in codominant, dominant, recessive, and overdominant models of inheritance via binary logistic regression and reported as odds ratio (OR) and 95% confidence interval (CI). Additional adjustment for potential confounders, such as age, body mass index (BMI), smoking, and time of the exposure in logistic regression enhanced the reliability of the obtained data. We, therefore, tested the effect of each SNP on each hematologic parameter among workers to assess for highly significant relationships. Linear regression was used to assess the effect of SNPs in *ATM*, *WRN*, and *H2AX* on the hematologic parameters count by adjustment for age, BMI, smoking, and time of the exposure. For each analysis, the most prevalent homozygous genotype was used as the reference group. Ultimately Pearson correlation coefficient was used to assess correlation of measured parameters with years of exposure and working hours. All *p*‐values were two‐sided and *p* < 0.05 was established as a statistically significant level. All data are presented on the Github page (https://github.com/samanehjafariroshan/Genetic‐Variations‐).

## 3 RESULTS

### 3.1 Characteristics of study subjects

In the current study, participants were male and all variables, including age, BMI, the length of exposure day, job history, and smoking habits, showed no significant differences in the case and control groups. The median age and BMI were 34 years (range 21–58) and 25.57 kg/$m^{2}$ (range 16.48–36.06), respectively. Moreover, subjects had 12 h (range 4–16) exposure duration and 7.5 years (range, 2–24) job history. Only 5.8% of the population had smoking experience. The characteristics of comparison groups are presented in Table 2.

**TABLE 2:** Characteristics of the BTEXs and other variables in two groups of the study participants

Variables	Case *n* = (141)	Control *n* = (152)	*p*‐value
Age (years)	34.25 ± 6.54	34.59 ± 5.58	0.60
BMI (kg/m^2^)	25.86 ± 3.37	25.97 ± 3.12	0.92
Length of exposure in a day (hours)	11.79 ± 1.43	11.66 ± 1.42	0.47
Job history (year)	7.46 ± 3.96	7.92 ± 4.34	0.36
Smoking: no (%)			
Yes	7 (5.2%)	10 (6.8%)	0.55
No	128 (94.8%)	136 (93.2%)	
BTEX‐exposure measurements (ppm)			
Benzene	0.61 ± 1.600	0.75 ± 1.647	0.56
Toluene	1.05 ± 2.100	1.02 ± 2.09	0.76
Ethylbenzene	1.45 ± 2.53	1.27 ± 2.70	0.47
o‐xylene	0.424 ± 1.31	0.399 ± 1.61	0.28
p‐xylene	2.46 ± 3.09	2.29 ± 3.01	0.62
M xylene	1.65 ± 2.44	1.81 ± 2.62	0.60

Note

Data are presented as mean ± SD or number (percentage); Student *t* test was used to determine the statistical significance of the differences in quantitative variables and the chi‐square test was used for categorical variables; *p*‐value < 0.05 was considered statistically significant

Abbreviations: BMI, body mass index; BTEX, benzene toluene ethylbenzene xylene.

### 3.2 BTEX exposure assessment data

The results of analyses showed that the mean arithmetic concentrations of benzene, toluene, and ethylbenzene in the study population were 0.75 (standard deviation ±6.05 ppm), 1.07 (standard deviation ±2.12 ppm), and 1.36 (standard deviation ±2.6 ppm), respectively. Xylene is principally presented as a mixture of ortho, para, and meta isomers and their means of concentrations were 0.42 (standard deviation ±1.46 ppm), 2.36 (standard deviation ±3.04 ppm), and 1.73 (standard deviation ±2.53 ppm), respectively. Both examined groups statistically showed no significant differences in the arithmetic means of BTEXs concentration in the breathing zones and results are summarized in Table 2.

### 3.3 Hematological data

Evaluating the blood parameters in case and control groups showed that the mean hemoglobin (HGB), hematocrit (HCT), mean corpuscular volume (MCV), mean corpuscular hemoglobin (MCH), mean corpuscular hemoglobin concentration (MCHC), and granulocyte counts of cases were significantly lower than those of the control group (*p* = 0.001, 0.049, 0.006, 0.002, 0.001, and 0.001, respectively), while the mean red cell distribution width (RDW) and lymphocyte counts in cases were significantly higher than those observed in the control group (*p* = 0.001 and 0.001, respectively). No significant difference in white blood cell count (WBC), red blood cell count (RBC), platelet (PLT) counts, and monocyte counts were observed between the two groups. Details are presented in Table 3.

**TABLE 3:** Data on hematological indices in case and control subjects

Hematological indices	Case *n* = (141)	Control *n* = (152)	*p*‐value
WBC (×10^9^/L)	8.15 ± 1.86	7.19 ± 1.86	0.701
RBC (×10^12^/L)	4.90 ± 0.390	4.84 ± 0.322	0.466
Hgb (g/dl)	14.74 ± 2.071	15.06 ± 0.815	**0.001**
HCT (%)	42.92 ± 4.64	43.57 ± 2.11	**0.049**
MCV (fL)	87.95 ± 10.28	90.05 ± 3.51	**0.006**
MCH (Pg)	30.22 ± 4.45	31.13 ± 1.38	**0.002**
MCHC (g/dl)	34.25 ± 1.54	34.53 ± 0.680	**0.001**
RDW (%)	12.17 ± 0.649	12.02 ± 0.598	**0.001**
PLT (×10^9^/L)	204.6 ± 36.69	210.3 ± 50.23	0.842
Lymphocytes (%)	46.15 ± 7.94	38.10 ± 6.80	**0.001**
Monocytes (%)	2.94 ± 8.33	3.83 ± 2.43	0.326
Granulocytes (%)	51.04 ± 7.94	58.05 ± 6.70	**0.001**

Note

Values are given as the mean ± standard deviation; Student *t* test for comparing the difference of hematological indices in the case and control groups was used; *p*‐value < 0.05 was considered statistically significant (in bold).

Abbreviations: HCT, hematocrit; Hgb, hemoglobin; MCH, mean corpuscular hemoglobin; MCHC, mean corpuscular hemoglobin concentration; MCV, mean corpuscular volume; PLT, platelet; RBC, red blood cell count; RDW, red cell distribution width; WBC, white blood cell count.

### 3.4 Associations of selected polymorphisms with the risk of abnormal hematological indices

Table 4 describes the distribution of alleles and genotypes of the *ATM*‐rs228589 A>T, *WRN*‐rs1800392 G>T, and *H2AX*‐rs7759 A>G SNPs in the study population. All SNP genotypes were in Hardy–Weinberg equilibrium among all of the examined participants (*p* > 0.05). The genotype frequency distributions of SNPs rs228589 and rs7759 were significantly different between cases and controls (*p* = 0.032, 0.003, respectively). In the case of *ATM*‐rs228589 A>T polymorphism, the T allele in the case group was significantly higher than the control group (*p* = 0.012). Also, heterozygous AT genotype and homozygous TT genotype of this SNP were more prevalent in the case group than that in the control group (AT 46.8% and TT 8/5% vs. AT 34.9% and TT 5.3%, *p* = 0.032). Moreover, it was observed that the G allele of *H2AX*‐rs7759 A>G has a higher frequency in the case group compared to the control group (*p* = 0.001). In line with this finding, cases had more heterozygous AG genotype and homozygous GG genotype than control individuals (AG 46.8% and GG 14.9% vs. AG 38.2% and GG 5.9%, *p* = 0.003). Considering the *WRN*‐rs1800392 G>T, the distribution of alleles and genotypes showed no significant difference between cases and controls (*p* = 0.37, 0.154, respectively).

**TABLE 4:** Genotype and allele distribution of the selected polymorphisms in study population groups

SNPs	Cases (*n* = 141)^a^ *n* (%)	Controls (*n* = 152)^a^ *n* (%)	*p*‐value	P‐HWE
rs228589A>T				
Genotypes				
AA	63 (44.7)	91 (59.9)		
AT	66 (46.8)	53 (34.9)	**0.032**	
TT	12 (8.5)	8 (5.3)		0.64
Alleles			**0.012**	
A	192 (68.1)	235 (77.3)		
T	90 (31.9)	69 (22.7)		
rs7759A>G				
Genotypes				
AA	54 (38.3)	85 (55.9)		
AG	66 (46.8)	58 (38.2)		
GG	21 (14.9)	9 (5.9)	**0.003**	0.76
Alleles				
A	174 (61.7)	228 (75)	**0.001**	
G	108 (38.3)	76 (25)		
rs1800392G>T				
Genotypes				
GG	60 (42.6)	76 (50)		
GT	62 (44)	61 (40.1)	0.377	
TT	19 (13.5)	15 (9.9)		0.44
Alleles				
G	182 (64.5)	213 (70.1)	0.154	
T	100 (35.5)	91 (29.9)		

Note

Data are presented as number and percentage; chi‐square test was applied; Bold values are statistically significant (*p* < 0.05).

Abbreviations: P‐HWE, *p* value for Hardy–Weinberg equilibriumSNPs, single‐nucleotide polymorphisms.

^a^ The genotyping was successful in 141 cases and 152 controls for rs228589, rs7759, and rs1800392 polymorphisms.

The possible associations of different genotypes of the studied SNPs with the risk of abnormal hematological patterns were also evaluated via different genetic models and the results are shown in Table 5. After the adjustment for confounders, such as age, BMI, smoking, and the length of exposure to BTEX, AT and TT genotypes of *ATM*‐rs228589 A>T showed a significant association with a risk of hematological abnormalities in the codominant model (AT vs. AA, OR: 1.829, 95% CI: 1.107–3.023, *p* = 0.018), dominant model (AT + TT vs. AA, OR: 1.890, 95% CI: 1.163–3.071, *p* = 0.010), and overdominant model (AT vs. AA + TT, OR: 1.680, 95% CI: 1.032–2.734, *p* = 0.037). There was a little variation in OR values for unadjusted results (Table 5). After the adjustment for the above various variables, genetic model assessments showed that GG and AG genotypes of the *H2AX*‐rs7759 A>G SNP were significantly associated with an increased risk of the abnormal pattern of hematological indices under the codominant model (GA vs. AA, OR: 2, 95% CI: 1.192–3.356, *p* = 0.009 & GG vs. AA, OR: 3.472, 95% CI: 1.451–8.305, *p* = 0.005), dominant model (AG + GG vs. AA, OR: 2.239, 95% CI: 1.371–3.657, *p* = 0.001), and recessive model (GG vs. AA + AG, OR: 2.594, 95% CI: 1.129–5.957, *p* = 0.025). For unadjusted analysis, the same trend was observed (Table 5). Regarding the *WRN*‐rs1800392 G>T, statistical evaluations did not show any significant results (Table 5).

**TABLE 5:** Association results between SNP genotypes and patterns of hematological parameters in multiple inheritance models

SNPs	Genotype models	Genotypes	Population groups		Unadjusted OR (95% CI)	*p*	Adjusted OR* (95% CI)	*p*
			Cases (*n* = 141)	Controls (*n* = 152)				
ATM‐rs228589 A>T	Codominant	A/A A/T T/T	63 (44.7%)	91 (59.9%)	1		1	
			66 (46.8%)	53 (34.9%)	1.799 (1.109–2.917)	**0.017**	1.829 (1.107–3.023)	**0.018**
			12 (8.5%)	8 (5.3%)	2.167 (0.838–5.605)	0.111	2.464 (0.88–6.899)	0.086
	Dominant	A/A A/T + T/T	63 (44.7%)	91 (59.9%)	1		1	
			78 (55.3%)	61 (40.1%)	1.847 (1.161–2.938)	**0.010**	1.890 (1.163–3.071)	**0.010**
	Recessive	A/A + A/T T/T	129 (91.5%)	144 (94.7%)	1		1	
			12 (8.5%)	8 (5.3%)	1.674 (0.664–4.225)	0.275	1.629 (0.622–4.265)	0.321
	Overdominant	A/A + T/T A/T	75 (53.2%)	99 (65.1%)	1		1	
			66 (46.8%)	53 (34.9%)	1.644 (1.028–2.629)	**0.038**	1.680 (1.032–2.734)	**0.037**
H2AX‐rs7759 A>G	Codominant	A/A A/G G/G	54 (38.3%)	85 (55.9%)	1		1	
			66 (46.8%)	58 (38.2%)	1.791 (1.097–2.926)	**0.020**	2 (1.192–3.356)	**0.009**
			21 (14.9%)	9 (5.9%)	3.673 (1.567–8.611)	**0.003**	3.472 (1.451–8.305)	**0.005**
	Dominant	A/A A/G + G/G	54 (38.3%)	85 (55.9%)	1		1	
			87 (61.7%)	67 (44.1%)	2.044 (1.282–3.260)	**0.003**	2.239 (1.371–3.657)	**0.001**
	Recessive	A/A + A/G G/G	120 (85.1%)	143 (94.1%)	1			
			21 (14.9%)	9 (5.9%)	2.781 (1.227–6.299)	**0.014**	2.594 (1.129–5.957)	**0.025**
	Overdominant	A/A + G/G A/G	75 (53.2%)	94 (61.8%)	1		1	
			66 (46.8%)	58 (38.2%)	1.426 (0.896–2.271)	0.135	1.582 (0.970–2.581)	0.066
WRN‐rs1800392 G>T	Codominant	G/G G/T T/T	60 (42.5%)	76 (50%)	1		1	
			62 (44%)	61 (40.1%)	1.287 (0.789–2.100)	0.312	1.427 (0.852–2.391)	0.177
			19 (13.5%)	15 (9.9%)	1.604 (0.753–3.420)	0.221	1.666 (0.775–3.584)	0.191
	Dominant	G/G G/T + T/T	60 (42.6%)	76 (50%)	1		1	
			81 (57.4%)	76 (50%)	1.350 (0.851–2.141)	0.202	1.496 (0.922–2.428)	0.103
	Recessive	G/G + G/T T/T	122 (86.5%)	137 (90.1%)	1		1	
			19 (13.5%)	15 (9.9%)	1.422 (0.693–2.921)	0.337	1.413 (0.684–2.917)	0.350
	Overdominant	G/G + T/T G/T	79 (56%)	91 (59.9%)	1		1	
			62 (44%)	61 (40.1%)	1.171 (0.736–1.863)	0.506	1.288 (0.791–2.097)	0.309

Note

Data are presented as number and percentage; Logistic regression model was applied; *p*‐value < 0.05 was considered statistically significant (in bold).

Abbreviations: ATM, ataxia telangiectasia mutated; CI, confidence interval; OR, odds ratio; SNPs, single‐nucleotide polymorphisms.

* Adjustment for age, BMI, smoking, and length of exposure to benzene.

Moreover, we independently and specifically analyzed the influence of individual target SNP genotypes on each of the hematological parameters in BTEX‐exposed workers. After adjusting age, BMI, smoking, and the length of exposure to BTEX by a linear regression model, we observed a significant reduction in the RBC index in carriers of heterozygous AT and homozygous TT for *ATM*‐rs228589 A>T (*p*‐trend = 0.010) (Table 6). The homozygous and heterozygous subjects for the G allele of *H2AX*‐rs7759 A>G showed a reduction in lymphocyte counts and increase in granulocyte counts (*p*‐trend = 0.010, *p*‐trend = 0.008, respectively) (Table 7). However, our data did not reveal any significant association between the *WRN*‐rs1800392 G>T genotypes and patterns of hematological indices (Table S1). Finally, we measured the correlation of hematological indices with years of exposure and working hours in cases group, and as illustrated in Table S2, there was a positive correlation between RBC and HCT with working hours |(*r* = 0.221, *p* < 0.011, *r* = 0.178, *p* = 0.043), respectively (Table S2).

**TABLE 6:** Genotypes of ATM‐rs228589 A>T polymorphism and their effects on the hematological parameters in the study population

Hematological parameters	ATM genotypes in controls (*n* = 152)			ATM genotypes in cases (*n* = 141)		
	TT (8)	AT (53)	AA (91)	TT (12)	AT (66)	AA (63)
WBC Mean ± SD	6.97 ± 1.831	6.98 ± 1.97	6.88 ± 1.62	7.105 ± 1.49	7.05 ± 1.51	6.38 ± 1.51
*p*‐value	Ref	0.915	0.464	Ref	0.830	0.489
*p*‐trend			0.874			0.200
RBC Mean ± SD	5.19 ± 0.640	4.96 ± 0.502	4.73 ± 0.343	4.98 ± 0.365	5.03 ± 0.363	5.02 ± 0.306
*p*‐value	Ref	**0.042**	**0.036**	Ref	0.179	0.278
*p*‐trend			**0.010**			0.788
HB Mean ± SD	14.84 ± 1.53	14.76 ± 1.54	14.35 ± 1.28	15.33 ± 0.815	15.31 ± 0.900	15.36 ± 0.850
*p*‐value	Ref	0.845	0.270	Ref	0.784	0.809
*p*‐trend			0.311			0.919
HCT Mean ± SD	44.12 ± 4.05	42.99 ± 3.09	43.09 ± 3.74	44.16 ± 2.21	44.27 ± 0.332	44.35 ± 0.500
*p*‐value	Ref	0.091	0.289	Ref	0.371	0.442
*p*‐trend			0.365			0.827
MCV Mean ± SD	85.26 ± 9.68	86.42 ± 9.92	88.55 ± 4.04	88.79 ± 3.77	88.23 ± 3.74	88.48 ± 4.98
*p*‐value	Ref	0.631	0.266	Ref	0.348	0.494
*p*‐trend			0.154			0.830
MCH Mean ± SD	28.99 ± 4.38	30.03 ± 3.39	30.60 ± 1.63	30.83 ± 1.65	30.53 ± 1.75	30.65 ± 1.78
*p*‐value	Ref	0.176	0.340	Ref	0.204	0.313
*p*‐trend			0.179			0.765
MCHC Mean ± SD	33.84 ± 1.90	34.28 ± 2.08	34.18 ± 1.01	34.71 ± 0.867	34.59 ± 0.984	34.66 ± 0.942
*p*‐value	Ref	0.212	0.563	Ref	0.262	0.358
*p*‐trend			0.586			0.873
RDW Mean ± SD	12.37 ± 1.00	12.42 ± 0.946	12.65 ± 0.856	12.10 ± 0.558	12.16 ± 0.573	12.07 ± 0.572
*p*‐value	Ref	0.630	0.225	Ref	0.560	0.676
*p*‐trend			0.362			0.884
PLT Mean ± SD	207.4 ± 52.9	210.6 ± 39.6	231.3 ± 46.1	210.9 ± 40.6	213.0 ± 38.2	217.0 ± 46.2
*p*‐value	Ref	0.941	0.285	Ref	0.592	0.627
*p*‐trend			0.108			0.682
LYM Mean ± SD	44.31 ± 11.89	44.77 ± 9.99	46.22 ± 8.74	38.11 ± 5.32	37.53 ± 6.41	36.06 ± 4.73
*p*‐value	Ref	0.883	0.445	Ref	0.827	0.445
*p*‐trend			0.576			0.330
MO Mean ± SD	2.92 ± 1.50	2.99 ± 1.72	2.30 ± 0.961	3.13 ± 1.93	3.01 ± 1.58	3.31 ± 1.12
*p*‐value	Ref	0.860	0.099	Ref	0.432	0.694
*p*‐trend			0.215			0.787
GR Mean ± SD	51.59 ± 11.36	50.99 ± 10.28	49.95 ± 8.55	58.09 ± 5.70	58.22 ± 7.46	58.73 ± 5.77
*p*‐value	Ref	0.815	0.515	Ref	0.992	0.931
*p*‐trend			0.628			0.785

Note

Data are presented as the mean ± standard deviation; Linear regression was applied; Bold values are statistically significant (*p* < 0.05).

Abbreviations: ATM, ataxia telangiectasia mutated; HCT, hematocrit; Hgb, hemoglobin; MCH, mean corpuscular hemoglobin; MCHC, mean corpuscular hemoglobin concentration; MCV, mean corpuscular volume; PLT, platelet; RBC, red blood cell count; RDW, red cell distribution width; WBC, white blood cell count.

**TABLE 7:** Genotypes of H2AX‐RS7759 A>G polymorphism and their effects on the hematological parameters in the study population

Hematological parameters	H2AX genotypes in cases (*n* = 141)			H2AX genotypes in controls (*n* = 152)		
	GG (9)	AG (58)	AA (85)	GG (21)	AG (66)	AA (54)
WBC Mean ± SD	6.852 ± 1.41	7.24 ± 1.98	6.42 ± 2.270	7.084 ± 1.626	7.02 ± 1.371	6.91 ± 1.366
*p*‐value	Ref	0.704	0.417	Ref	0.728	0.828
*p*‐trend			0.173			0.933
RBC Mean ± SD	5.05 ± 0.609	5.11 ± 0.573	4.82 ± 0.433	4.96 ± 0.370	5.04 ± 0.355	5.06 ± 0.300
*p*‐value	Ref	0.442	0.152	Ref	0.207	0.514
*p*‐trend			0.133			0.382
HB Mean ± SD	14.91 ± 1.35	14.73 ± 1.49	14.45 ± 1.93	15.24 ± 0.875	15.48 ± 0.798	15.32 ± 0.70
*p*‐value	Ref	0.199	0.229		0.117	0.746
*p*‐trend			0.499	Ref		0.187
HCT Mean ± SD	43.76 ± 2.84	43.58 ± 4.07	42.61 ± 3.95	43.98 ± 2.31	44.61 ± 2.21	43.83 ± 1.73
*p*‐value	Ref	0.269	0.141	Ref	0.180	0.836
*p*‐trend			0.459			0.230
MCV Mean ± SD	87.43 ± 7.92	85.13 ± 9.01	86.21 ± 13.83	88.79 ± 3.86	88.57 ± 3.73	86.68 ± 3.69
*p*‐value	Ref	0.245	0.490	Ref	0.491	0.161
*p*‐trend			0.422			0.290
MCH Mean ± SD	29.87 ± 3.75	29.28 ± 3.92	30.01 ± 3.58	30.78 ± 1.73	30.76 ± 1.65	29.88 ± 1.41
*p*‐value	Ref	0.633	0.971	Ref	0.858	0.194
*p*‐trend			0.615			0.316
MCHC Mean ± SD	34.04 ± 1.76	34.20 ± 2.10	33.78 ± 1.85	34.64 ± 0.889	34.73 ± 0.957	34.50 ± 0.832
*p*‐value	Ref	0.636	0.548	Ref	0.411	0.761
*p*‐trend			0.671			0.732
RDW Mean ± SD	12.48 ± 0.889	12.28 ± 0.663	12.69 ± 1.67	12.12 ± 0.557	12.07 ± 0.567	12.43 ± 0.519
*p*‐value	Ref	0.714	0.552	Ref	0.554	0.183
*p*‐trend			0.209			0.573
PLT Mean ± SD	205.8 ± 44.8	215.3 ± 46.4	210.4 ± 52.6	209.8 ± 38.3	216.5 ± 42.3	203.5 ± .38.7
*p*‐value	Ref	0.659	0.800	Ref	0.796	0.612
*p*‐trend			0.541			0.497
LYM Mean ± SD	47.35 ± 11.04	44.29 ± 8.69	39.10 ± 13.62	38.28 ± 5.41	37.38 ± 5.98	36.01 ± 6.35
*p*‐value	Ref	**0.001**	**0.003**	Ref	0.346	0.232
*p*‐trend			**0. 010**			0.408
MO Mean ± SD	2.67 ± 1.09	3.14 ± 1.82	2.65 ± 1.74	2.93 ± 1.66	3.38 ± 1.95	2.86 ± 1.61
*p*‐value	Ref	0.811	0.782	Ref	0.168	0.961
*p*‐trend			0.168			0.306
GR Mean ± SD	48.13 ± 10.56	52.10 ± 8.75	56.12 ± 13.62	58.10 ± 6.22	57.94 ± 6.53	60.32 ± 6.38
*p*‐value	Ref	**0.001**	**0.003**	Ref	0.886	0.305
*p*‐trend			**0.008**			0.573

Note

Data are presented as the mean ± standard deviation; Linear regression was applied; Bold values are statistically significant (*p* < 0.05).

Abbreviations: ATM, ataxia telangiectasia mutated; HCT, hematocrit; Hgb, hemoglobin; MCH, mean corpuscular hemoglobin; MCHC, mean corpuscular hemoglobin concentration; MCV, mean corpuscular volume; PLT, platelet; RBC, red blood cell count; RDW, red cell distribution width; WBC, white blood cell count.

## 4 DISCUSSION

It is now clear that the level of susceptibility of individuals to the effects of toxicants such as BTEXs is variable, often due to genetic differences.^27^, ^30^, ^31^, ^34^, ^35^ Revealing such a genetic predisposition can be very important for exposure risk assessment and early detection and prevention of related diseases in susceptible individuals.^1^, ^30^, ^31^, ^36^ Blood cells and bone marrow are the main cells and tissue that show the highest level of sensitivity and manifestations due to the toxic effects of BTEX compounds,^4^ but the responses vary among exposed individuals, signifying the existence of an underlying genetic predisposition.^13^, ^27^, ^37^ Previous investigations have revealed the role of genetic variations in some candidate genes involved in DNA damage and repair in susceptibility to harmful hematotoxic effects of BTEX compounds.^13^, ^26^ BTEX metabolites and their reactive metabolites can cause genomic damage, including DBS lesions,^2^, ^3^ thus resulting in cell cycle arrest, stopping the replication of damaged DNA, and triggering DBS repair responses.^16^, ^18^ It is, therefore, biologically plausible to suppose that polymorphisms of loci controlling the DBS repair pathway may contribute to *t* determining individual susceptibility to harmful effects of BTEXs on blood cells.

The current study indicated that functional genetic variants, *ATM*‐rs228589 A>T and *H2AX*‐rs7759 A>G, within two key loci acting in the DNA DSB repair system were significantly related to some abnormal patterns of hematological indices in workers exposed to BTEX. Epidemiologic researches indicate that BTEX, particularly benzene, behave as hematotoxicants and carcinogens, and their occupational exposure is linked to the causation of some human health conditions including hematological abnormalities.^6^, ^38^ In this regard, benzene exposure in mild form is linked to the abnormality in RBC counts, hemoglobin concentration, leukocyte counts, MCHC, and platelets^38^, ^39^ and in severe form, exposed subjects show an increased risk of aplastic anemia, myelodysplastic syndromes, and leukemia.^6^, ^7^ Likewise, there are some reports on the exposure to toluene, ethylbenzene, and xylene with the development of hematological abnormality.^38^ Nevertheless, it has been shown that individuals may respond differently to these toxins under similar exposure conditions, and early clues suggest the role of individual genetic differences.^25^, ^27^, ^34^

Our analysis showed that the T allele of *ATM* ‐rs228589 A>T was significantly more prevalent in cases than the control individuals. In addition, the genetic model analysis revealed a significant association of T‐bearing genotypes in the dominant model (AT + TT vs. AA), overdominant model (AT vs. AA + TT), and codominant model (AT vs. AA) with a high risk of abnormal hematological indices. Specifically, AT and TT genotypes were associated with the reduced RBC counts. These significant findings were observed in both adjusted and unadjusted conditions suggesting that individuals carrying AT and TT genotypes may be prone to RBC abnormalities as the specific hematotoxicity caused by exposure to BTEX. The *ATM* gene encodes a serine/threonine kinase activity, which controls the maintenance of genomic integrity via promoting the repair of DSB damages, and regulation of the cell cycle and apoptosis.^16^, ^40^ The DSB repair pathway seems to be a key defense machine against the BTEX‐induced genotoxicity and hematotoxicity.^3^, ^13^, ^24^, ^41^ Of note, the product of the *ATM* gene plays a very important role in the function of blood cells and bone marrow‐derived cells, and its defect is linked with serious abnormalities of the hematopoietic cells.^24^, ^42^ Once the DNA DSB repair system is induced, the ATM phosphorylates the key H2AX protein for transducing repair signal to downstream effectors at DSB mutation sites.^18^, ^40^

Notably, the rs228589 variant is within the promoter region of the *ATM* gene and may lead to the appearance of specific phenotypes via altering its expression and cellular function.^43^ For example, evidence indicates that it is associated with a decrease in DNA repair capacity^16^ and the risk of some human diseases.^16^, ^44^ In line with our results, Wang et al reported that DNA damage response capacity in polycyclic aromatic hydrocarbons (PAHs)‐exposed workers was significantly lower in individuals carrying AT and AT + TT genotypes of rs228589 compared to subjects carrying AA genotype.^16^ According to data from an epidemiological investigation, the T allele of rs228589 is linked with a higher risk of breast cancer development in Jewish non‐Ashkenazi women.^43^ Therefore, although the functional role of this polymorphism in the promoter of the ATM gene has not been clarified, it is thought that its T allele may alter *ATM* activity in the DSB repair by altering its expression and, ultimately, reducing genomic integrity. As a result, such an *ATM* gene variation may determine an individual's susceptibility to BTEX‐induced blood disorders.

Additionally, we discovered that the G allele of *H2AX*‐rs7759 A>G SNP was more frequent in exposed subjects with abnormal hematological patterns than in the normal exposed subjects. Moreover, the analysis of genetic models showed that its GG and AG genotypes were significantly related to abnormalities of hematological indices under the codominant model (GA vs. AA, & GG vs. AA), dominant model (AG + GG vs. AA), and recessive model (GG vs. AA + AG). Furthermore, it was demonstrated that the G allele of *H2AX*‐rs7759 A>G was associated with increased granulocyte counts and decreased lymphocyte counts. Likewise, the result was significantly the same in this case after considerations in both adjusted and unadjusted levels. As mentioned in previous sections, *H2AX* is the key gene in the DSB repair pathway and its rapid phosphorylation in serine 139 by ATM kinase is the main signal for the recruitment of DSB‐related proteins such as NBS1, TP53BP1, and BRCA1, during repair responses in DSB sites.^17^, ^18^ It is also observed that inactivating mutations in *H2AX* lead to genome instability and mutation repair defects.^45^, ^46^ Interestingly, it is claimed that due to the conservation nature of the *H2AX* coding region, genetic variants outside the coding region may be responsible for the development of most cases of *H2AX*‐related multifactorial diseases by interaction with environmental factors. Especially, this variant is located in the promoter of *H2AX* and therefore, may be linked with a functional change in the protein product by altering the efficiency of gene expression.^47^ In line with the importance of our results, previously published data by Sun et al. have shown that the G allele of *H2AX*‐rs7759 is associated with DNA damage levels in individuals exposed to particulate matter 2.5 (PM2.5).^48^ Moreover, previous studies have indicated that the G allele of *H2AX*‐rs7759 is related to an increased risk of breast cancer.^49^ Accordingly, our observations supportively suggest that the *H2AX*‐rs7759 polymorphism and its G allele‐related genotypes could be one of the key genetic targets in influencing the effects of BTEX chemicals and increase the susceptibility of individuals exposed to the BTEX harmful effects, especially abnormalities in lymphocytes and granulocytes, through altering the mutation repair capacity.

In summary, this is the first research that provided evidence for the relationships between *ATM* rs228589 A>T and *H2AX*‐rs7759 A>G polymorphisms and hematotoxicity among BTEX‐exposed workers in an Iranian subpopulation. The obtained results support our hypothesis that selected genetic polymorphisms in target key genes regulating the DSB repair may confer susceptibility to hematotoxicity in workers exposed to BTEX. In our specific results, *ATM*‐rs228589 A>T seemed to influence RBC while *H2AX*‐rs7759 A>G altered granulocytes and lymphocytes, proposing the biological effects of these gene variants may be cell‐specific. Such findings may help clarify the underlying mechanisms of BTEX‐induced DNA damage, such as DBS lesions, and developing new screening approaches in the near future for identifying vulnerable subjects that are at a high risk of hematotoxicity due to BTEX exposure. Other strengths of the present study were the homogeneous ethnic background of the population and the well‐documented workplace exposure history for BTEX. It should be noted that our study also had certain limitations, including the issue that it could be done on a larger population with other various ethnic groups. Therefore, further larger studies are needed to verify our findings and determine the exact biological function of these polymorphisms and their effects on gene expression and linkage disequilibrium with the other functional SNPs to explore the effect of these and related SNPs on blood cells and the risk of hematotoxicity in individuals exposed to BTEXs as hematotoxins.

## CONFLICTS OF INTEREST

The authors declare that they have no competing interests.

## AUTHOR CONTRIBUTIONS

S‐JR and AD: Project development/management, Data curation, Data analysis, Visualization, Manuscript writing/editing, and Funding acquisition. YM and SR‐H: Project management, Data analysis/curation, and Interpretation of the results. DS: Data analysis and Manuscript writing/review.

## Supporting information

Fig S1

Fig S2

Fig S3

Table S1‐S2

Supplementary Material

## Data Availability

The data that support the findings of this study are available from the corresponding author upon reasonable request.
