# Supplementary figures and images for: Genetic variations in ATM and H2AX loci contribute to risk of hematological abnormalities in individuals exposed to BTEX chemicals
Source: J Clin Lab Anal. 2022 Mar 2;36(4):e24321. doi: 10.1002/jcla.24321 (PMC8993635; doi:10.1002/jcla.24321)

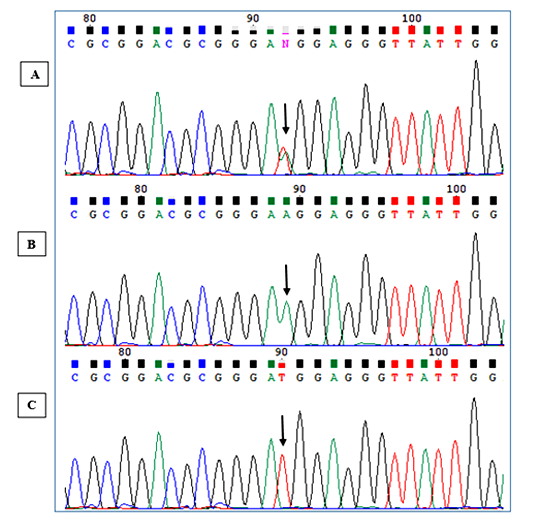

Supplement: Supplementary file 1 — Fig S1 [file JCLA-36-e24321-s004.tif]

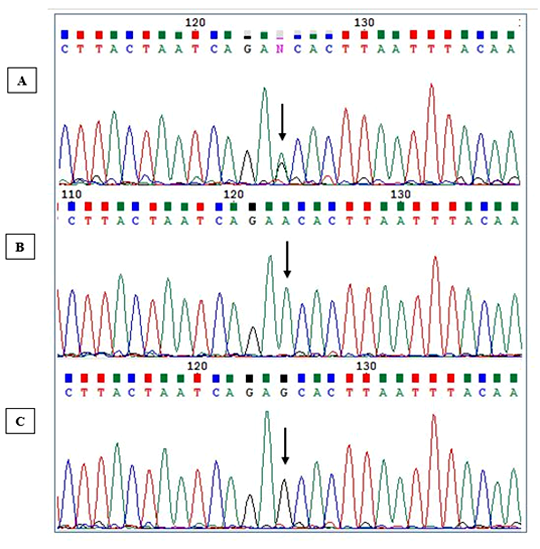

Supplement: Supplementary file 2 — Fig S2 [file JCLA-36-e24321-s005.tif]

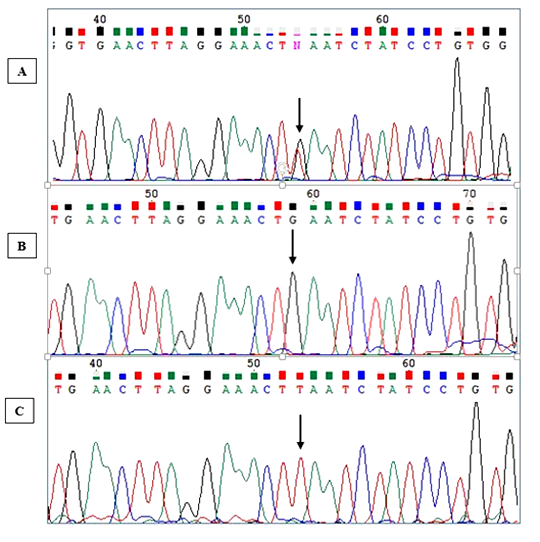

Supplement: Supplementary file 3 — Fig S3 [file JCLA-36-e24321-s002.tif]
