# Supplementary material for: Genetic variations in ATM and H2AX loci contribute to risk of hematological abnormalities in individuals exposed to BTEX chemicals
Source: J Clin Lab Anal. 2022 Mar 2;36(4):e24321. doi: 10.1002/jcla.24321 (PMC8993635; doi:10.1002/jcla.24321)
Supplement: Supplementary file 4 — Table S1‐S2 [file JCLA-36-e24321-s003.docx]

**Table S1.** Genotypes of WRN-RS1300892 G>T polymorphism and their effects on the hematological parameters in the study population

| **WRN genotypes in controls (n=152)** | | | **WRN genotypes in cases (n=141)** | | | **Hematological parameters** |
| --- | --- | --- | --- | --- | --- | --- |
| TT(15) | GT(61) | GG(76) | TT(19) | GT(62) | GG(60) |  |
| 7.28±1.37 | 7.21±1.61 | 6.87±1.45 | 6.90±2.30 | 7.006±1.78 | 6.90±1.77 | WBC Mean± SD  P-Value  P-trend |
| 0.374 | 0.189 | Ref | 0.978 | 0.978 | Ref |  |
| 0.341 |  |  | 0.977 |  |  |  |
| 5.00±0.346 | 5.00±0.367 | 5.00±0.363 | 5.23±0.726 | 5.00±0.544 | 5.03±0. 549 | RBC Mean± SD  P-Value  P-trend |
| 0.793 | 0.323 | Ref | 0.170 | 0.977 | Ref |  |
| 0.974 |  |  | 0.192 |  |  |  |
| 15.16±1.05 | 15.25±0.783 | 15.41±0.843 | 14.68±1.71 | 14.60±1.55 | 14.95±1.41 | HB Mean± SD |
| 0.473 | 0.539 | Ref | 0.431 | 0.109 | Ref | P-Value |
| 0.308 |  |  | 0.504 |  |  | P-trend |
| 43.72±2.13 | 44.10±2.27 | 44.40±2.27 | 43.63±3.68 | 43.25±4.31 | 44.73±2.76 | HCT Mean± SD |
| 0.459 | 0.996 | Ref | 0.967 | 0.411 | Ref | P-Value |
| 0.287 |  |  | 0.916 |  |  | P-trend |
| 87.40±3.29 | 88.49±4.10 | 88.87±3.65 | 84.52±10.53 | 85.38±10.86 | 87.51±7.35 | MCV Mean± SD |
| 0.139 | 0.214 | Ref | 0.146 | 0.107 | Ref | P-Value |
| 0.175 |  |  | 0.234 |  |  | P-trend |
| 30.31±1.70 | 30.64±1.79 | 30.85±1.60 | 28.54±4.90 | 29.71±3.72 | 29.85±3.48 | MCH Mean± SD |
| 0.208 | 0.125 | Ref | 0.160 | 0.488 | Ref | P-Value |
| 0.257 |  |  | 0.193 |  |  | P-trend |
| 34.65±1.29 | 34.62±0.958 | 34.71±0.781 | 33.58±2.24 | 34.15±1.77 | 34. 16±2.00 | MCHC Mean± SD |
| 0.780 | 0.209 | Ref | 0.218 | 0.635 | Ref | P-Value |
| 0.821 |  |  | 0.262 |  |  | P-trend |
| 12.22±0.488 | 12.11±0.628 | 12.11±0.521 | 12.71±1.42 | 12.39±1.02 | 12.36±.0.682 | RDW Mean± SD |
| 0.436 | 0.640 | Ref | 0.197 | 0.604 | Ref | P-Value |
| 0.498 |  |  | 0.170 |  |  | P-trend |
| 214.2±28.3 | 211.7±41.4 | 211.7±40.9 | 207.4 ±58.7 | 215.7±41.6 | 207.1±47.7 | PLT Mean± SD |
| 0.866 | 0.923 | Ref | 0.900 | 0.586 | Ref | P-Value |
| 0.827 |  |  | 0.980 |  |  | P-trend |
| 39.53±3.74 | 36.95±6.29 | 38.14±5.43 | 41.81±11.26 | 46.02±11.53 | 44.22±9.63 | LYM Mean± SD |
| 0.339 | 0.405 | Ref | 0.516 | 0.359 | Ref | P-Value |
| 0.389 |  |  | 0.393 |  |  | P-trend |
| 2.95±2.23 | 2.93±1.46 | 3.26±1.91 | 2.50±1.37 | 3.04±1.78 | 2.89±1.41 | MO Mean± SD |
| 0.427 | 0.105 | Ref | 0.359 | 0.464 | Ref | P-Value |
| 0.389 |  |  | 0.345 |  |  | P-trend |
| 56.50±3.68 | 59.16±6.89 | 57.71±6.22 | 53.11±10.42 | 49.96±10.75 | 51.79±10.52 | GR Mean± SD |
| 0.427 | 0.348 | Ref | 0.770 | 0.330 | Ref | P-Value |
| 0.501 |  |  | 0.637 |  |  | P-trend |

Note: Data are presented as the mean ± standard deviation; Linear regression was applied; Bold values are statistically significant (P < .05). Abbreviations: ATM, ataxia telangiectasia mutated; WBC, white blood cell count; RBC, red blood cell count; Hgb, hemoglobin; HCT, hematocrit; MCV, mean corpuscular volume; MCH, mean corpuscular hemoglobin; MCHC, mean corpuscular hemoglobin concentration; RDW, red cell distribution width; PLT, platelet.

**Table** **S2**. Correlation of hematological indices with years of exposure and working hours for cases group

| **Hematological indices** | **Years of exposure** | | **Working hours** | |
| --- | --- | --- | --- | --- |
|  | **r** | **P-Value** | **r** | **P-Value** |
| WBC | -0.137 | 0.120 | -0.020 | 0.819 |
| RBC | -0.086 | 0.327 | 0.221 | **0.011** |
| Hgb | -0.009 | 0.917 | 0.135 | 0.125 |
| HCT | -0.100 | 0.258 | 0.178 | **0.043** |
| MCV | 0.024 | 0.784 | -0.096 | 0.275 |
| MCH | 0.030 | 0.730 | -0.100 | 0.258 |
| MCHC | 0.044 | 0.621 | -0.037 | 0.673 |
| RDW | -0.048 | 0.585 | 0.093 | 0.292 |
| Platelets | -0.036 | 0.682 | 0.009 | 0.916 |
| Lymphocytes | -0.037 | 0.674 | 0.068 | 0.445 |
| Monocytes | 0.086 | 0.330 | 0.066 | 0.455 |
| Granulocytes | 0.082 | 0.356 | -0.096 | 0.277 |

Note: Data are presented as numbers; Pearson Correlation test was applied; Bold values are statistically significant level (P < .05). Abbreviations: r, Pearson correlation coefficient; ATM, ataxia telangiectasia mutated; WBC, white blood cell count; RBC, red blood cell count; Hgb, hemoglobin; HCT, hematocrit; MCV, mean corpuscular volume; MCH, mean corpuscular hemoglobin; MCHC, mean corpuscular hemoglobin concentration; RDW, red cell distribution width; PLT, platelet.
