# Supplementary material for: Genetic variations in ATM and H2AX loci contribute to risk of hematological abnormalities in individuals exposed to BTEX chemicals
Source: J Clin Lab Anal. 2022 Mar 2;36(4):e24321. doi: 10.1002/jcla.24321 (PMC8993635; doi:10.1002/jcla.24321)
Supplement: Supplementary file 5 — Supplementary Material [file JCLA-36-e24321-s001.docx]

**Figure S1.** DNA sequencing chromatograms of ATM-RS228589A>T. The arrow point designates the corresponding SNP: **(A)** Heterozygous form of rs228589 (A/T); **(B)** Wild-type rs228589 (A/A); **(C)** Homozygous genotype of rs228589 (T/T).

**Figure S2.** DNA sequencing chromatograms of H2AX-RS7759A>G. The arrow point shows the corresponding SNP: **(A)** Heterozygous pattern of rs7759 (A/G); **(B)** Wild-type genotype of rs7759 (A/A); **(C)** Homozygous variant genotype of rs228589 (G/G).

**Figure S3.** DNA sequencing chromatograms of WRN-RS1300892G>T: The arrow point specifies the corresponding SNP: **(A)** Heterozygous mutant genotype of rs1800392 (G/T); **(B)** Wild-type form of rs1800392 (G/G); **(C)** Homozygous mutant form of rs1800392 (T/T).
